# First‐line crizotinib versus platinum‐pemetrexed chemotherapy in patients with advanced ROS1‐rearranged non‐small‐cell lung cancer

**DOI:** 10.1002/cam4.2972

**Published:** 2020-03-13

**Authors:** Lan Shen, Tan Qiang, Ziming Li, Ding Ding, Yongfeng Yu, Shun Lu

**Affiliations:** ^1^ Shanghai Lung Cancer Center Shanghai Chest Hospital Shanghai Jiao Tong University Shanghai China; ^2^ Department of Oncology Johns Hopkins Medical Institutions Baltimore MD USA

**Keywords:** crizotinib, first‐line chemotherapy, non‐small‐cell lung cancer (NSCLC), pemetrexed, *ROS1* rearrangement

## Abstract

**Objectives:**

Food and Drug Administration (FDA) approved crizotinib for advanced ROS1‐rearranged (*ROS1+*) non‐small‐cell lung cancer (NSCLC) patients due to a single‐arm study PROFILE 1001. However, there is no direct comparison between crizotinib and platinum‐pemetrexed chemotherapy.

**Materials and methods:**

Clinical data of advanced *ROS1+*NSCLC patients treated with first‐line crizotinib or platinum‐pemetrexed chemotherapy between August 2010 and December 2017 were analyzed.

**Results:**

Seventy‐seven patients were eligible, including 30 (39.0%) in the crizotinib group and 47 (61.0%) in the platinum‐pemetrexed chemotherapy group. The median follow‐up was 28.1 months (95% confidence interval [CI]: 19.2‐39.0). The objective response rate (ORR) of crizotinib (86.7%, 95% CI: 73.3‐96.7) was higher than that of platinum‐pemetrexed chemotherapy (44.7%, 95% CI: 29.8‐57.4, *P* < .001). The disease control rate (DCR) was 96.7% (95% CI: 90.0‐100) in the crizotinib group and 85.1% (95% CI: 74.5‐95.7) in the chemotherapy group (*P* = .140). Significantly longer progression‐free survival (PFS) was observed in the patients treated with crizotinib (18.4 months, 95% CI: 6.4‐30.3) versus platinum‐pemetrexed chemotherapy (8.6 months, 95% CI: 6.9‐10.3, *P* < .001). Overall survival (OS) was also compared between the two groups and no significant difference was seen (Not reach vs 28.4 months [95% CI: 20.7‐36.0], *P* = .176). Notably, a total of 37 patients have treatment crossover after the failure of first‐line treatment. Among those patients, difference in OS was not statistically significant between seven patients who have given first‐line crizotinib (38.6 months, 95% CI: 0‐81.0) and 30 patients who have given platinum‐pemetrexed chemotherapy initially (32.8 months, 95% CI: 11.9‐53.8, *P* = .805).

**Conclusions:**

Our results suggested that first‐line crizotinib had higher ORR and longer PFS than platinum‐pemetrexed chemotherapy in patients with advanced *ROS1+*NSCLC, but the differences were not observed for OS.

## Graphical abstract

This is the first study to compare crizotinib with chemotherapy in ROS1+NSCLC. Crizotinib was superior to platinum‐pemetrexed chemotherapy as an initial treatment.

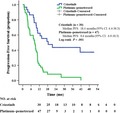

## 1 INTRODUCTION

ROS1 rearrangement (*ROS1+*) is an actionable driver mutation that occurs in 1% to 2% of patients with non‐small‐cell lung cancer (NSCLC).^1^, ^2^, ^3^, ^4^ Crizotinib, an oral ALK/MET/ROS1 inhibitor, has shown marked antitumor activities in *ROS1+*NSCLC. The PROFILE 1001 study of crizotinib included a subgroup of 50 patients with *ROS1+*NSCLC. The objective response rate (ORR) of these patients was 72% and median progression‐free survival (PFS) was 19.2 months after the administration of crizotinib.^5^ Based on this single‐arm result, the US Food and Drug Administration approved crizotinib as first‐targeted drug to treat advanced *ROS1+*NSCLC.^6^ More recently, in a large phase 2 study (OO‐1201) which enrolled 127 East Asian patients with advanced *ROS1+*NSCLC, crizotinib produced an ORR of 71.7% with 17 complete responses (CR) and 74 partial responses (PR), a median duration of response (DOR) of 19.7 months, and a median PFS of 15.9 months. These findings provided additional clinical evidence of crizotinib treatment as a valid strategy in patients with ROS1 rearrangement.^7^

Although the development of targeted medication has improved treatment responses and survival outcomes for patients with advanced *ROS1+*NSCLC, traditionally cytotoxic chemotherapy remains the other standard treatment. As a multitargeted antineoplastic agent, pemetrexed is active in inhibiting the folate‐dependent enzyme that is necessary for the synthesis of pyrimidine and purine. In 2008, pemetrexed in combination with cisplatin or carboplatin has been approved for the standard first‐line chemotherapy of non‐squamous NSCLC depending on the data of JMDB. Previous studies showed that *ROS1+*patients could benefit from pemetrexed‐based chemotherapy.^8^, ^9^, ^10^, ^11^, ^12^ Chen et al found that patients with ROS1 fusion had a better ORR (57.9%), higher disease control rate (DCR; 89.5%), and longer PFS (7.5 months) of pemetrexed‐based chemotherapy compared with patients harboring other driver mutations.^8^ In a retrospective research by Drilon et al, 10 patients with *ROS1+*NSCLC were treated with pemetrexed‐based systemic therapy and achieved 23 months in the median PFS.^13^

However, the efficacy of first‐line crizotinib versus platinum‐pemetrexed chemotherapy in advanced *ROS1+*NSCLC currently remains unknown. All the clinical trials that demonstrate a robust antitumor efficacy with crizotinib in advanced *ROS1+*NSCLC patients are single‐arm studies,^5^, ^7^, ^14^ including PROFILE1001 and OO‐1201 study. No prospective or retrospective studies have been designed to directly compare crizotinib treatment with standard chemotherapy in those patients due to its low incidence. In this study, we performed a retrospective analysis to directly compare the clinical efficacy of crizotinib with platinum‐pemetrexed chemotherapy as initial treatment in advanced *ROS1+*NSCLC patients.

## 2 MATERIALS AND METHODS

### 2.1 Study population

**FIGURE 1:**
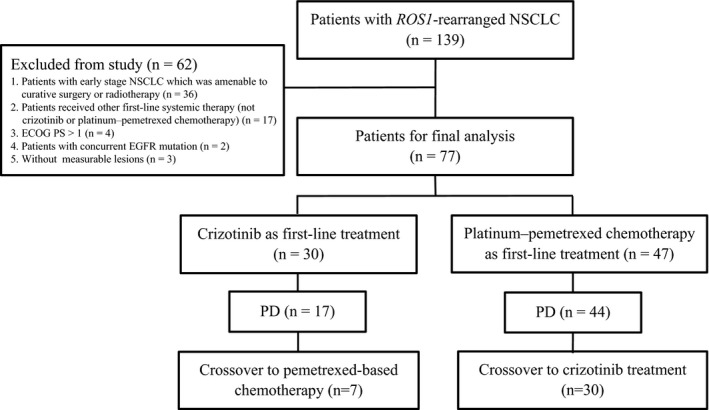
Flow diagram illustrating study populations. ECOG PS, Eastern Cooperative Oncology Group performance status; NSCLC, non‐small‐cell lung cancer; PD, progressive disease

Between August 2010 and December 2017, 139 consecutive patients with *ROS1+*NSCLC were treated at Shanghai Chest Hospital. Of these, patients were enrolled if they fulfilled the following criteria: locally advanced or metastatic NSCLC; histologically confirmed NSCLC; Eastern Cooperative Oncology Group performance status (ECOG PS): 0‐1; harboring ROS1 rearrangement with EGFR wild type and ALK negative; received crizotinib or platinum‐pemetrexed chemotherapy (pemetrexed plus cisplatin or carboplatin with or without bevacizumab) in the first‐line setting; have measurable lesions per Response Evaluation Criteria in Solid Tumors (RECIST) version 1.1. Finally, a total of 77 patients were enrolled for final analysis, including 30 patients in the crizotinib group and 47 patients in the platinum‐pemetrexed chemotherapy group. The remaining 62 patients were excluded from this analysis: 36 patients had early stage NSCLC which was amenable to curative surgery or radiotherapy, 17 patients received other first‐line systemic therapy (not crizotinib or platinum‐pemetrexed chemotherapy), four patients had ECOG PS > 1, two patients had concurrent EGFR mutation, and three patients lacked measurable lesions (Figure 1).

### 2.2 Data collection

We reviewed medical records of all patients to collect data on clinical characteristics (age, gender, histological diagnosis, smoking history, stage, distant metastasis organ, gene status, survival data, and information about treatments). All patients underwent tumor assessment every two cycles, or when they had significant clinical signs of progression. Tumor assessments were performed by dedicated radiologists according to the RECIST version 1.1.^15^ Radiologic response to therapy was classified as CR, PR, stable disease (SD), or progressive disease (PD). ORR was defined as the percentage of patients with the best response of CR or PR, and DCR was defined as the percentage of patients with the best response of CR, PR, or SD. Toxicity was summarized on the basis of the Common Terminology Criteria for Adverse Events, version 4.0 (CTCAE v4.0). PFS was defined as the time from the first dose of crizotinib or platinum‐pemetrexed chemotherapy to the first documentation of objective tumor progression or death, and those who were alive without evidence of PD were censored at the last follow‐up date. Overall survival (OS) was defined as the time from the first dose of crizotinib or platinum‐pemetrexed chemotherapy to the date of death or the last follow‐up. The last follow‐up date was 30 January 2019. This study gained approval by the ethics committees of the Shanghai Chest Hospital.

### 2.3 Identification of ROS1 rearrangement

ROS1 rearrangement was tested by real‐time polymerase chain reaction (RT‐PCR) using a ROS1 fusion gene detection kit which can detect 14 ROS1 fusions (Amoy Diagnostics, Xiamen, China).^16^ ROS1 fusion partners were confirmed by Sanger sequencing or next generation sequencing (NGS).

### 2.4 Statistical analyses

Categorical variables were summarized and compared via chi‐squared test or Fisher's exact test. PFS and OS were estimated by the Kaplan‐Meier curve and then compared using the log‐rank test. Multivariate analysis was carried out using Cox proportional hazard model. All statistical tests were two‐sided and *P *< .05 were considered statistically significant. The computer software SPSS version 19.0 was used to perform the analyses.

## 3 RESULTS

### 3.1 Patient characteristics

Overall, a total of 77 patients harboring ROS1 rearrangement satisfied the inclusion criteria to be included in this study (Figure 1). The median age of 77 patients was 53 years (range 29‐78 years) with a slight female predominance (64.9%, 50/77). Most of the patients were non‐smokers (80.5%, 62/77) and diagnosed as lung adenocarcinoma (98.7%, 76/77; Table 1). ROS1 fusion partners were confirmed in 35 patients (45.5%, 35/77) with the following distribution: CD74‐ROS1 (48.6%, 17/35), EZR‐ROS1 (25.7%, 9/35), SLC34A2‐ROS1 (11.4%, 4/35), SDC4‐ROS1 (11.4%, 4/35), and TPM3‐ROS1 (2.9%, 1/35).

**TABLE 1:** Clinicopathologic characteristics of patients (n = 77)

Characteristic	All patients (n = 77)	Crizotinib (n = 30)	Platinum‐pemetrexed (n = 47)	*P* value
Age, years				
Median	53	51.5	55	
Range	29‐78	29‐78	33‐76	
Age distribution, n (%)				.309
≤65 y	61 (79.2)	22(73.3)	39 (83.0)	
>65 y	16 (20.8)	8 (26.7)	8 (17.0)	
Gender, n (%)				.457
Male	27 (35.1)	9 (30.0)	18 (38.3)	
Female	50 (64.9)	21 (70.0)	29 (61.7)	
Smoking status, n (%)				.618
Never	62 (80.5)	25 (83.3)	37 (78.7)	
Ever/current	15 (19.5)	5 (16.7)	10 (21.3)	
Histology, n (%)				1.000
Adenocarcinoma	76 (98.7)	30 (100.0)	46 (97.9)	
Non‐adenocarcinoma	1 (1.3)	0 (0)	1 (2.1)	
Stage,n (%)				1.000
IIIB‐IIIC	8 (10.4)	3 (10.0)	5 (10.6)	
IVA‐IVB	69 (89.6)	27 (90.0)	42(89.4)	
Postoperative recurrence, n (%)				.618
Yes	15 (19.5)	5 (16.7)	10 (21.3)	
No	62 (80.5)	25 (83.3)	37 (78.7)	
Brain metastases				.111
No	61 (79.2)	21 (70.0)	40 (85.1)	
Yes	16 (20.8)	9 (30.0)	7 (14.9)	
*ROS1* fusion partner				.164
CD74‐*ROS1*	17 (22.1)	10 (33.3)	7 (14.9)	
Non CD74‐*ROS1*	18 (23.4)	6 (20.0)	12 (25.5)	
Unknown	42 (54.5)	14 (46.7)	28 (59.6)	

Baseline clinicopathologic characteristics of patients treated with crizotinib (n = 30) were compared with those treated with platinum‐pemetrexed chemotherapy (n = 47) as first‐line treatment (Table 1). No significant difference between the crizotinib and chemotherapy groups was found in age, gender, smoking status, histology, stage, postoperative recurrence, brain metastases (BM), and ROS1 fusion partner at baseline (Table 1). Among the 47 patients in the chemotherapy group, 11 patients (23.4%) received platinum‐pemetrexed therapy in combination with bevacizumab. A total of 22 patients were treated with pemetrexed maintenance after four cycles of induction therapy.

### 3.2 Overall antitumor efficacy

**FIGURE 2:**
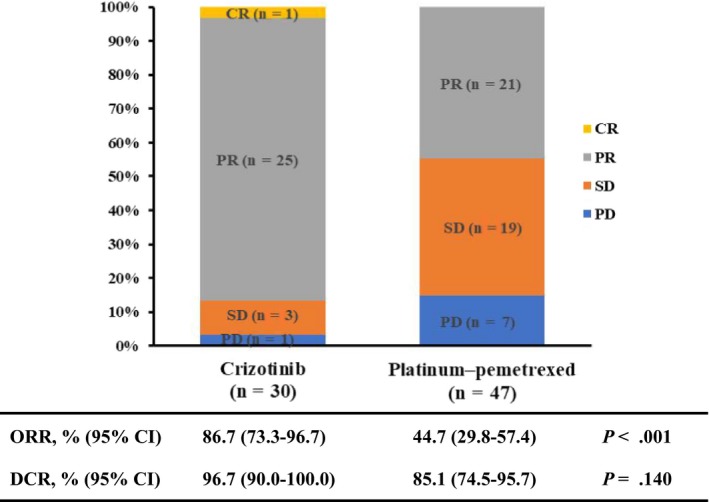
Best tumor response to crizotinib or platinum‐pemetrexed chemotherapy as first‐line treatment. CI, confidence interval; CR, complete response; DCR, disease control rate; ORR, overall response rate; PD, progressive disease; PR, partial response; SD, stable disease

Among the 30 patients treated with crizotinib, one patient (3.3%) had CR, 25 (83.3%) had PR, three (10.0%) had SD, and one (3.3%) had PD as their best response. Among the 47 patients treated with platinum‐pemetrexed chemotherapy, 21 patients (44.7%) had PR, 19 (40.4%) had SD, and seven (14.9%) had PD as their best response. The ORR in the crizotinib group was 86.7% (95% confidence interval [CI]: 73.3‐96.7), which was statistically higher than that in the platinum‐pemetrexed chemotherapy group (44.7%, 95% CI: 29.8‐57.4, *P* < .001).The DCR was 96.7% (95% CI: 90.0‐100.0) with crizotinib treatment and 85.1% (95% CI: 74.5‐95.7) with platinum‐pemetrexed chemotherapy (*P* = .140; Figure 2).

**FIGURE 3:**
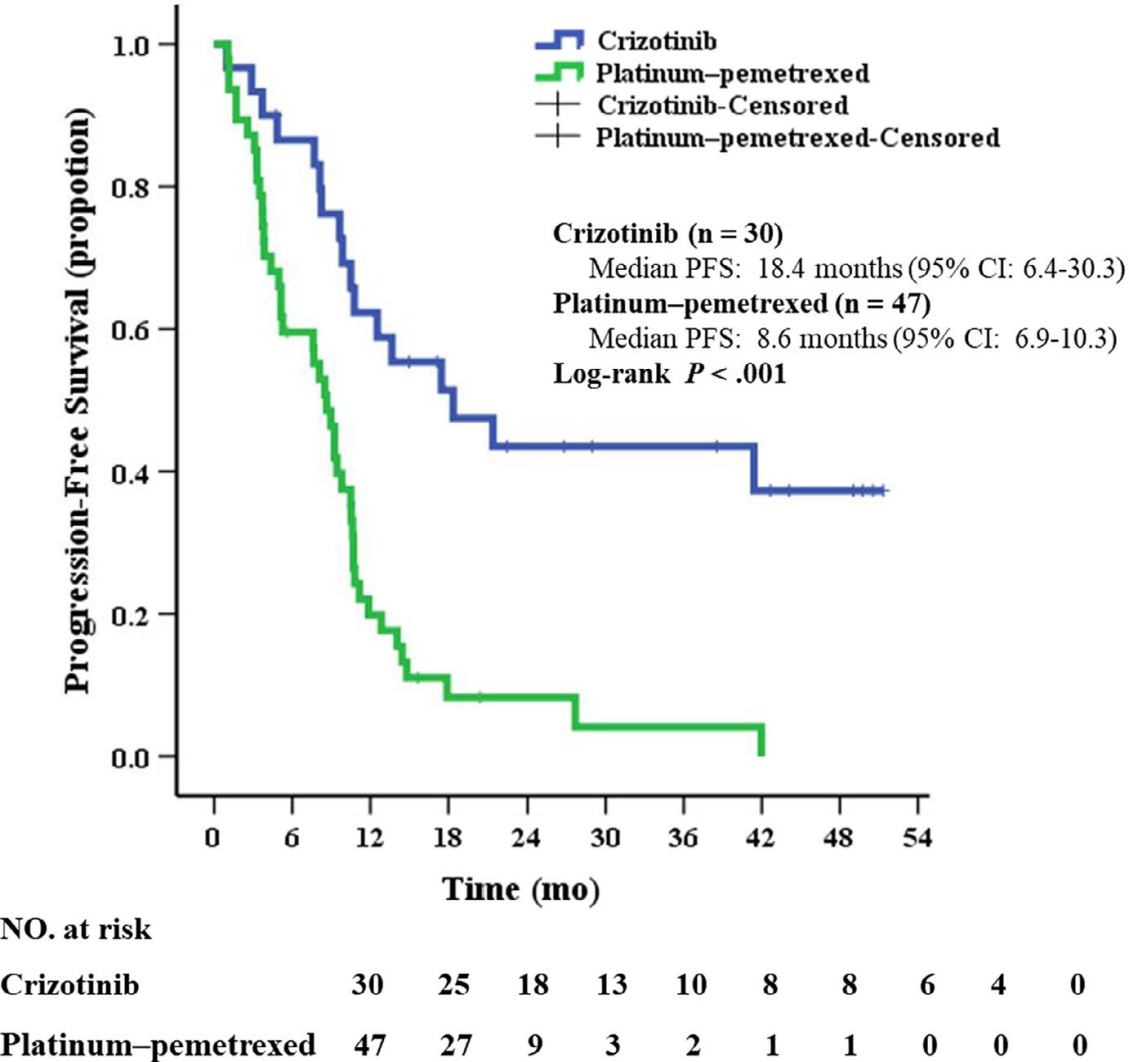
Kaplan‐Meier curves of progression‐free survival in patients treated with crizotinib or platinum‐pemetrexed chemotherapy as first‐line treatment. CI, confidence interval; PFS, progression‐free survival

The median follow‐up period of this study was 28.1 months (95% CI: 19.2‐39.0) for entire patients. Three patients (3.9%, 3/77) were lost to follow‐up. Progression events occurred in 17 patients (56.7%, 17/30) in the crizotinib group and 44 patients (93.6%, 44/47) in the chemotherapy group. The median PFS was 18.4 months (95% CI: 6.4‐30.3) for the patients treated with crizotinib, which was significantly longer than that of 8.6 months (95% CI: 6.9‐10.3) for those treated with platinum‐pemetrexed chemotherapy group (*P* < .001; Figure 3). While for those who chose to receive platinum‐pemetrexed chemotherapy as their initial treatment (n = 47), no difference in PFS was found whether the regimen comprised bevacizumab (9.0 months, 95% CI: 6.7‐11.3) or not (8.1 months, 95% CI: 3.6‐12.6, *P* = .746). Multivariable analysis indicated that first‐line crizotinib treatment (hazard ratio [HR]: 0.199, 95% CI: 0.105‐ 0.378, *P* < .001) and absence of BM (HR: 0.261, 95% CI: 0.132‐0.515, *P* < .001) were independently associated with the improved PFS (Table 2).

**TABLE 2:** Univariate and multivariate analysis of progression‐free survival and overall survival in all patients (n = 77)

Variable	Progression‐free survival			Overall survival		
	Univariate analysis	Multivariate analysis		Univariate analysis	Multivariate analysis	
	*P* value	HR (95% CI)	*P* value	*P* value	HR (95% CI)	*P* value
Age						
≤65 years versus > 65 years	.590	1.110 (0.578‐2.131)	.755	.828	1.029 (0.421‐2.514)	.950
Gender						
Male versus female	.479	0.700 (0.342‐1.433)	.330	.803	0.909 (0.383‐2.160)	.830
Smoking status						
Never versus ever/current	.639	1.202 (0.497‐2.909)	.683	.806	1.106 (0.345‐3.547)	.865
Stage						
IIIB‐IIIC versus IVA‐IVB	.783	1.051 (0.425‐2.600)	.914	.637	0.968 (0.278‐3.368)	.959
Brain metastases						
No versus yes	.012	0.261 (0.132‐0.515)	<.001	.002	0.313 (0.145‐0.673)	.003
First‐line treatment						
Crizotinib versus platinum‐pemetrexed	<.001	0.199 (0.105‐0.378)	<.001	.176	0.565 (0.269‐1.189)	.133

Abbreviations: CI, confidence interval; HR, hazard ratio.

**FIGURE 4:**
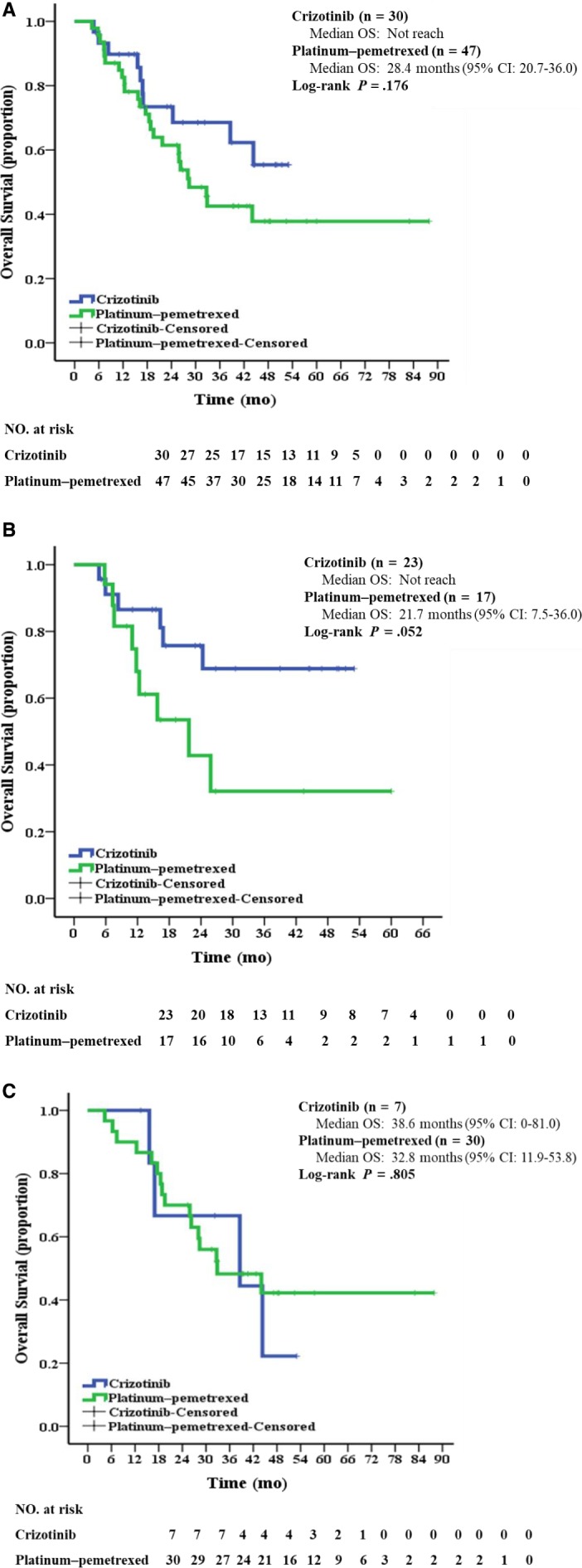
Kaplan‐Meier curves of overall survival (A) in all patients treated with crizotinib or platinum‐pemetrexed chemotherapy as first‐line treatment (B) in patients who had not treatment crossover (C) in patients who had treatment crossover. CI, confidence interval; OS, overall survival

At the time of data cutoff, 35 patients (45.5%, 35/77) had died: 10 (33.3%, 10/30) in the crizotinib group and 25 (53.2%, 25/47) in the platinum‐pemetrexed chemotherapy group. Median OS between the crizotinib group (Not reach) and chemotherapy group (28.4 months, 95% CI: 20.7‐36.0) was not significantly different (*P* = .176; Figure 4A). Multivariable analysis showed that the absence of BM (HR: 0.313, 95% CI: 0.145‐0.673; *P* = .003) was independently significant factors for OS benefit (Table 2).

Notably, a total of 37 patients have treatment crossover after the failure of first‐line treatment. Seven patients chose to receive first‐line crizotinib and subsequently crossed over to pemetrexed‐based therapy after disease progression, while 30 patients received first‐line platinum‐pemetrexed chemotherapy before crizotinib treatment. To further address whether similar OS might have been confounded by treatment crossover, we performed a subgroup analysis. Among patients who had no treatment crossover (n = 40), those who used crizotinib in the first‐line setting tended to have numerically longer median OS than those who received platinum‐pemetrexed therapy, however, this difference was not statistically significant (Not reach vs 21.7 months [95% CI: 7.5‐36.0], *P* = .052; Figure 4B). While for those who had treatment crossover (n = 37), difference in median OS was not statistically significant between seven patients who have given first‐line crizotinib (38.6 months, 95% CI: 0‐81.0) and 30 patients who have given platinum‐pemetrexed chemotherapy initially (32.8 months, 95% CI: 11.9‐53.8, *P* = .805; Figure 4C).

### 3.3 Sites of disease progression and subsequent therapies

At the last follow‐up, 61 of 77 patients (79.2%) had PD. Extracranial PD alone was the most common type of PD, both in the crizotinib (33.3%, 10/30) and in the chemotherapy group (83.0%, 39/47). Among the 30 patients in the crizotinib groups, seven patients (23.3%) had intracranial PD and five of them were with isolated intracranial PD. Among the 47 patients in the chemotherapy groups, five patients had intracranial PD (10.6%) and all of them were combined with extracranial progression. While there was a higher percentage of patients with intracranial PD during treatment in the crizotinib group, it was not statistically significant (23.3% vs 10.6%, *P* = .134). Among the 61 patients without BM at baseline, the development of BM occurred in seven patients (11.5%, 7/61) and no significant difference was found between the two groups (14.3% vs 10.0%, *P* = .683).

After disease progression, four patients (23.5%, 4/17) in the crizotinib group received supportive treatment and 13 patients (76.5%, 13/17) received subsequent anticancer treatment, including radiotherapy, crizotinib beyond PD, next generation ROS1‐TKI, pemetrexed‐based therapy, and non‐pemetrexed‐based chemotherapy. In the platinum‐pemetrexed chemotherapy group, seven patients (15.9%, 7/44) received supportive treatment after PD and 37 patients (84.1%, 37/44) received subsequent anticancer treatment, including crizotinib or other ROS1‐TKIs, non‐pemetrexed‐based chemotherapy, pemetrexed re‐challenge, and PD‐1/PD‐L1 inhibitors. Three patients with isolated intracranial PD continued to take crizotinib after brain radiotherapy and showed an additional PFS of 11, 16, and 26 weeks. One patient in the chemotherapy group received pemetrexed re‐challenge and experienced PD after 4.8 months.

### 3.4 Safety

Treatment‐related adverse events (AEs) of 77 patients were shown in Supplemental Table 1. The most common AEs in the crizotinib group (n = 30) were alanine aminotransferase (ALT) elevation (53.3%), aspartate aminotransferase (AST) elevation (43.3%), and nausea (36.7%). Dose reductions or temporary interruption occurred in five patients (16.7%, 5/30) due to the Grade 3 or 4 AEs, and no one interrupted crizotinib target therapy due to treatment‐related AEs.

The most common AEs reported in the chemotherapy group (n = 47) were leukopenia (40.4%), neutropenia (31.9%), and fatigue (31.9%). Grade 3 or 4 AEs occurred in eight patients (17.0%, 8/47), and most of them were able to be relieved by symptomatic therapies.

### 3.5 Discussions

This is the first study to directly and systemically compare the therapeutic efficacy of crizotinib with platinum‐pemetrexed chemotherapy and determine which regimen is better in treating advanced *ROS1+*NSCLC. Our findings indicated that advanced *ROS1+*NSCLC patients who received crizotinib had better response rates and a longer PFS than those received platinum‐pemetrexed chemotherapy as first‐line treatment. But these apparent differences in ORR and PFS were not observed for OS.

Among our cohort of 30 *ROS1+*NSCLC patients who received crizotinib treatment, the median PFS was 18.4 months, which was similar to the results of PROFILE 1001^5^ and was longer than the PFS reported by Wu et al^7^ Of note, the ORR reported here was much higher than that previous reported in PROFILE 1001^5^ and OO‐1201.^7^ Given that these previous studies did not breakout the clinical effect by line of crizotinib therapy, the higher ORR may contribute to all the patients in our study given crizotinib in the first‐line. Recently, OS data of PROFILE 1001 were updated after a long‐term follow‐up (62.6 months) and it was remarkably extended to 51.4 months.^17^ OS observed with crizotinib in present study was not reached, probably owing to the relatively short follow‐up (28.1months) and low death rate (33.3%).

In this study, we observed that *ROS1+*patients had an ORR of 44.7% and a PFS of 8.6 months when they received first‐line platinum‐pemetrexed chemotherapy. The data were better than the results reported in previous clinical trials enrolling general population of NSCLC patients.^18^, ^19^, ^20^ These findings revealed that patients with *ROS1+*NSCLC were likely to be more responsive to pemetrexed‐based chemotherapy, which were broadly in line with results of previous studies conducted in *ROS1+*NSCLC.^8^, ^9^, ^10^, ^21^ However, the benefit of platinum‐pemetrexed chemotherapy was less than that of crizotinib treatment. As shown in this study, *ROS1+*NSCLC patients receiving first‐line crizotinib had statistically higher ORR and longer PFS than patients using platinum‐pemetrexed chemotherapy initially.

Nonetheless, we found no significant difference in OS between crizotinib and chemotherapy groups. As well as other studies,^22^, ^23^ the subsequent treatment strategies after disease progression might confound the true efficacy of the initial first‐line treatment on OS. In our study, we found that the crossover rate of patients from the chemotherapy group to the crizotinib group was higher, which was the most plausible explanation for the lack of OS benefit despite PFS and ORR advantage with crizotinib therapy. More importantly, the patients tended to have inferior OS when received initial therapy with platinum‐pemetrexed chemotherapy and did not cross over to crizotinib, as compared with those who received crizotinib initially but not switched to pemetrexed. Similarly, shorter OS (20.3 months) in the *ROS1+*patients who accepted pemetrexed but without any target therapy was also observed in a study from South Korea.^21^ These data reflected the fact that the addition of crizotinib may contribute to the trend toward improving OS in patients with *ROS1+*NSCLC. Despite its proven efficacy, many patients opted to receive platinum‐pemetrexed chemotherapy as their first choice, considering that delay of crizotinib treatment probably relieves their economic burden. In present study, we sought to learn whether the sequence in which the therapy was performed first affected the OS of *ROS1+*patients. Among the 37 *ROS1+*patients who had crossover after initial treatment failure, OS was not statistically different whether crizotinib or chemotherapy was the first‐line therapy. Our results showed that at least first‐line treatment of *ROS1+*NSCLC with platinum‐pemetrexed chemotherapy might not decrease OS as long as crizotinib is available as the further‐line treatment. Note that only seven patients crossed over to pemetrexed‐based therapy after crizotinib failure, the result that we did not find significant difference between these two groups may be limited by the current sample size. The power to detect the true difference will be improved when we are able to accumulate more patients in the future.

The safety profiles of crizotinib and platinum‐pemetrexed chemotherapy reported here were consistent with that reported previously. Elevation of ALT and AST levels were the most AEs observed with crizotinib, whereas leukopenia and fatigue occurred frequently in patients treated with chemotherapy. In general, the incidence of Grade 3 or 4 AEs was low in both groups. In addition, the patient‐reported quality of life (QOL) should be taken into consideration when making the choice of the first‐line therapy for *ROS1+*NSCLC. Patients tended to have an improvement in QOL and symptoms control during crizotinib treatment in the OO‐1201 study.^7^ However, these data were absence of a comparator arm. There is a limitation that QOL data during treatment in this study were not collected due to its retrospective nature. Further studies that investigate the patient‐reported QOL between the target therapy and chemotherapy may be of interest.

Despite the robust anti‐tumor activity of crizotinib against advanced *ROS1+*NSCLC, brain remained a frequent site of initial crizotinib failure, occurring in 23.3% of patients and most of them were isolated intracranial PD. As systemic therapies with crizotinib could have a better control of extracranial disease, BM is a major barrier to the successful long‐term treatment of patients with *ROS1+*NSCLC, probably because of poor penetration of crizotinib into the central nervous system. Thus, regular brain imaging during crizotinib treatment may be considered and next generation ROS1 inhibitors are needed to manage or prevent intracranial progression for *ROS1+*NSCLC patients.^24^ In integrated analysis of STARTRK‐1/‐2 and ALKA‐372‐001, 23 *ROS1+*NSCLC patients with baseline BM were treated with next generation ROS1 inhibitor‐Entrectinib. Overall, intracranial responses (PR and CR) were noted in 55% (11/20) of patients with measurable brain lesion and intracranial DOR was 12.9 months. In addition, lorlatinib and repotrectinib (TPX‐0005) also have demonstrated intracranial activity against ROS1.^25^ Of note, the proportion of BM under chemotherapy treatment was 10.6% and all of them had extracranial progression meanwhile. Extracranial PD represented the predominant pattern of failure in chemotherapy group in the current study. The impact of crizotinib or pemetrexed‐based therapy against BM in *ROS1+*patients needs further investigation.

There are some limitations of our study. First, on account of low incidence of *ROS1+*NSCLC, the sample size of this study was limited. Second, it was a retrospective and single‐institution study, and its findings therefore need to be further validated by multi‐institution studies or in larger patient population in a prospective manner. Third, some patients in the chemotherapy group did not receive continuous pemetrexed maintenance after successful induction therapy and therefore might not appreciate the full therapeutic benefit.

In conclusion, this study showed first‐line crizotinib prolonged PFS, increased response rates in *ROS1+*NSCLC patients compared with platinum‐pemetrexed chemotherapy. However, treatment‐related differences were not apparent for OS.

## 4 PRIOR PRESENTATION

The abstract of this study was presented as a poster at the 2019 Annual Meeting of ASCO on 31 May 2019 to 4 June 2019 in Chicago.

## AUTHORS' CONTRIBUTIONS

Lan Shen, Tan Qiang, and Shun Lu conceived and designed the work. Lan Shen, Tan Qiang, Ziming Li, and Yongfeng Yu collected and assembled the data. Lan Shen, Tan Qiang, Ding Ding, and Shun Lu contributed to the data analysis and interpretation. All the authors contributed to the manuscript writing and read and approved the final manuscript.

## Supporting information

Table S1

## Data Availability

The data that support the findings of this study are available from the corresponding author upon reasonable request. But these data are not publicly available because of patient privacy or ethical concerns.
