# Supplementary material for: First‐line crizotinib versus platinum‐pemetrexed chemotherapy in patients with advanced ROS1‐rearranged non‐small‐cell lung cancer
Source: Cancer Med. 2020 Mar 13;9(10):3310–8. doi: 10.1002/cam4.2972 (PMC7221427; doi:10.1002/cam4.2972)
Supplement: Supplementary file 1 — Table S1 [file CAM4-9-3310-s001.docx]

| **Supplemental Table 1**. Summary of adverse events (n = 77) | | | | |
| --- | --- | --- | --- | --- |
| Adverse Events | Crizotinib (n = 30)^#^ | | Platinum–pemetrexed (n = 47) ^#^ | |
|  | All Grades | Grade ≥ 3 | All Grades | Grade ≥ 3 |
| Leukopenia | 5(16.7) | 1 (3.3) | 19(40.4) | 5 (10.6) |
| Neutropenia | 3(10.0) | 1(3.3) | 15 (31.9) | 3 (6.4) |
| Anemia | 3(10.0) | 0 | 11(23.4) | 1 (2.1) |
| Alanine aminotransferase elevation | 16(53.3) | 3(10.0) | 8 (17.0) | 0 |
| Aspartate aminotransferase elevation | 13(43.3) | 0 | 5 (10.6) | 0 |
| Creatine kinase-MB elevation | 10(33.3) | 1(3.3) | 1 (2.1) | 0 |
| Blood creatinine elevation | 2(6.7) | 0 | 3 (6.4) | 0 |
| Fatigue | 2(6.7) | 0 | 15 (31.9) | 2(4.3) |
| Nausea | 11(36.7) | 0 | 11 (23.4) | 1 (2.1) |
| Vomiting | 6 (20.0) | 0 | 7 (14.9) | 0 |
| Decreased appetite | 6(20.0) | 0 | 17(36.2) | 0 |
| Diarrhea | 4(13.3) | 0 | 4 (8.5) | 0 |
| Visual disturbance | 5(16.7) | 0 | 0 | 0 |
| Peripheral edema | 5(16.7) | 0 | 0 | 0 |
| Rash | 1(3.3) | 0 | 2(4.3) | 0 |
| Sinus bradycardia | 3(10.0) | 1(3.3) | 0 | 0 |
| ^#^ Data are number of patients (%) | | | | |
